# Abusive Supervision and Creativity: Investigating the Moderating Role of Performance Improvement Attribution and the Mediating Role of Psychological Availability

**DOI:** 10.3389/fpsyg.2021.658743

**Published:** 2021-06-21

**Authors:** Can Wang, Yongchang Wei, Xuan Zhao, Xuan Zhang, Ye Peng

**Affiliations:** School of Business Administration, Zhongnan University of Economics and Law, Wuhan, China

**Keywords:** abusive supervision, employee creativity, psychological availability, performance improvement attribution, conservation of resource theory

## Abstract

The existing studies have indicated that abusive supervision affects creativity; however, the specific impact mechanism is still unclear due to the uncertain relationship between leadership and employee creativity. Based on the resource perspective, this study examines the influence of abusive supervision on creativity through psychological availability (PA) and the moderating of this mediation by performance improvement attribution (PIA). Based on a survey of 234 employees', the hypotheses have been tested and the results reveal that abusive supervision had a detrimental effect on employee creativity partially mediated by employee PA, and employees' PIA moderated the mediation. This study offers new insights into the mechanisms associated with the relationship between abusive supervision and creativity.

## 1. Introduction

With the in-depth integration of “Internet +” and “big data” with the economy and society, the change of internal and external environment intensifies the competition between enterprises, leading increasing pressure on leaders. Leaders would constantly transfer pressure to employees during work, which may increase the occurrence frequency of abusive supervision in enterprises. This abusive supervision would affect employees' ability to access resources which in turn will affect employees' creativity. Although some studies have shown that abusive supervision can affect employees' creativity (Liu et al., 2016b; Shen et al., 2020a,b), the specific impact mechanism needs to be explored further due to the complex relationship between leadership and employee creativity. Therefore, it is necessary to investigate the impact mechanism of abusive supervision on creativity to improve employees' creativity.

How to improve the creativity of employees has become the focus of enterprise management and the existing studies have explored various factors that will influence employees' creativity. Based on creativity interaction model by Woodman et al. (1993), personality traits (Oldham and Cummings, 1996), intrinsic motivation (Zhang and Bartol, 2010; Grant and Berry, 2011; Zhang et al., 2014b), self-efficacy (Tierney and Farmer, 2002; Liao et al., 2010; Zhang and Zhou, 2014; Huang et al., 2016; Liu et al., 2016a) have been proved to have important effects on individual creativity. Besides the above individual factors, some studies suggest that organizational factors can also affect individual creativity, such as job characteristics (Liu et al., 2011), leadership style (Zhang and Bartol, 2010; Zhang and Zhou, 2014; Dong et al., 2015; Gu et al., 2015; Zhang et al., 2018), colleague support (Zhou and George, 2001) and workplace rejection (Amabile and Pratt, 2016; Kwan et al., 2018). However, most previous literature has generally focused on positive factors, while the research concerning negative behaviors such as abusive supervision is limited (Liao et al., 2018). Qu et al. (2015) pointed out that future studies should further explore the impact of negative leadership behaviors on employees' creativity and study its mechanism. As one of the important manifestations of the negative leadership behavior of managers, abusive supervision will have an important on employees' creativity (Shen et al., 2020b).

Abusive supervision refers to employees' perceived emotional and psychological hostility toward their subordinates from leaders (Tepper, 2000; Zhang and Bednall, 2016). Typical abusive supervision behaviors include criticizing, accusing and insulting employees, humiliating employees in public, cold violence, restricting employees' access to effective information, and frequently mentioning past failures and mistakes (Tepper, 2000; Yu et al., 2018). By reviewing previous literature, this study concluded that most existing studies on the relationship between abusive supervision and employees' creativity and innovation followed three paths including cognition (Zhao et al., 2013; Liu et al., 2016b), emotion (Restubog et al., 2011; Whitman et al., 2013; Simon et al., 2015; Han et al., 2017; Oh and Farh, 2017), and motivation (Zhang and Zhou, 2014; Zhang et al., 2014a; Schaubroeck et al., 2015). Creative behavior, which is an activity beyond routine work, has high uncertainty, high implied risk and the possibility of failure and requires individuals to invest a large amount of resources (Binyamin and Carmeli, 2010). Thus, it is necessary to investigate how abusive supervision affects employee creativity from the resource perspective; however, there is no research explore the influence of abusive supervision on creativity based on relative theory.

The conservation of resource theory explains the exchange of resources between individuals and surroundings. Psychological availability (PA) is an individual's perception of the physical, emotional and cognitive resources, which reveals the process of exchanging resources between individuals and the environment. High psychological availability indicates that individuals have abundant physiological, emotional and cognitive resources to utilize, which means more resources can be invested in creative activities. On the contrary, the lower the psychological availability means the less available resources individual has, thus individuals will take a more cautious attitude on resources. Some studies have found that abusive supervision can increase employees' psychological pressure (Whitman et al., 2013; Nandkeolyar et al., 2014; Han et al., 2017), which leads to the decline of individual psychological availability and induces individual awareness of resource conservation. Therefore, this study explores how abusive supervision influence employee creativity by considering the mediating role of PA.

According to the conservation of resource theory, employees will invest fewer resources in their work when they are subjected to negative leadership. However, not all employees are like this. The communication between leaders and employees is a process of mutual attribution. In other words, employees' behavior can be influenced by psychological attribution. Different leves of performance improvement attribution will affect employees' different mental states, which will lead to make different behaviors and invest different resources (Tepper, 2000; Liao et al., 2010; Oh and Farh, 2017). When employees have a high-performance improvement attribution, they will tend to regard the abuse of the leader as an incentive and encouragement, and will devote more resources to creative activities. On the contrary, when employees have a low-performance improvement attribution, they will tend to regard the leader's abuse as a kind of scolding and criticism, and will spend less resources for creative activities. Thus, the effect of abusive supervision on creativity through employees' psychological availability may be influenced by employees' performance improvement attribution. However, so far as we know the effect has not been investigated in previous studies. Therefore, this study proposes that performance improvement attribution is a potential moderator of the relationship between abusive supervision and psychological availability.

The contributions of this research include the following aspects. First, the critical contribution of this study is that we re-examine the relationship between abusive supervision and employee creativity in the Chinese context from the conservation of resource perspective. Second, our study explores the mechanisms of abusive supervision and employee creativity by addressing the role of employees' psychological availability from a conservation of resources perspective. This new perspective may explain the decreasing creativity observed by employees who experience abusive supervision. Third, by examining the moderate role of performance improvement attribution on the relationship between abusive supervision and employee creativity, our research contributes is to guide employees to establish positive attribution awareness, which is helpful to creative activities.

## 2. Theory and Hypotheses

This section provides a theoretical basis for exploring the impact of abusive supervision on employees' creativity. Based on the conservation of resource theory, this study developed a conceptual framework and proposed related hypotheses. The conceptual framework will be leveraged to further explore the influential factors through empirical research.

### 2.1. Conservation of Resource Theory

Hobfoll (1989) proposed the conservation of resource theory (COR), which reveals the behavior of individuals under stressful situations, and describes the interaction of resources between individuals and the social environment. Some studies believe that anything which helps achieve a goal can be called a resource, such as material or conditional resources, constructive resources, social support, and energy resources, and personal characteristics (Shirom, 2003; Xanthopoulou et al., 2007; Hobfoll Stevan, 2011; Quinn et al., 2012; Kroon et al., 2015). In recent years, COR has been successfully applied in various fields to confirm its value (Li and Chih, 2020; Liu et al., 2020; Mao et al., 2020). It has been extended from the study of individual behavior to employee burnout and work-family balance.

In addition to conservation of resource theory, some studies have used social exchange theory (Bandura, 1977, 1978; Shen et al., 2020b), psychological safety theory and social identification theory (Liu et al., 2016b; Zhu and Zhang, 2019) to study the relationship between abusive supervision and creativity. However, we know that creativity is an activity outside the scope of work that requires an individual to invest many resources. The occurrence of abusive supervision will greatly reduce the output of individual resources. Meanwhile, psychological availability is the individual's perception and evaluation of the availability of their psychological resources. Therefore, from the perspective of resource conservation, the mechanism among these factors can be better studied.

Due to the previous reasons, based on the principle of resource conservation in resource conservation theory, a theoretical framework will be established for explaining the mechanism of abusive supervision's influence on employees' creativity, and demonstrating the role of psychological availability and performance improvement attribution in this process. The research results will be helpful in understanding leadership behavior and also provide reference measures for managers and organizations.

### 2.2. Research Hypotheses

**Figure 1:**
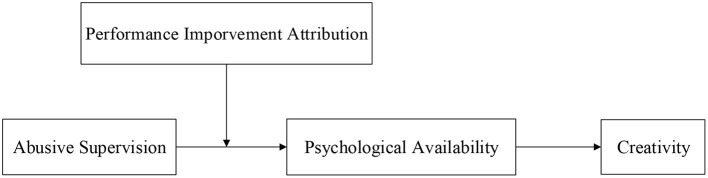
A research framework.

The following hypotheses mainly study the impact of abusive supervision on employees' creativity. Based on the Conservation of Resource Theory, we introduce psychological availability as an intermediary variable, and performance improvement attribution as a moderating variable to analyze the process of the mechanism. The research framework is shown in Figure 1.

#### 2.2.1. Abusive Supervision and Employees Creativity

Baumeister et al. (2001) pointed out that compared with the positive external environment, individuals are more sensitive to negative external environment reactions, and their attitudes and behaviors are also more susceptible to the negative environment. In an organization, employees' creativity means that individuals propose novel, practical ideas and suggestions for products, services, or programs, introduce new working methods and so on Amabile (1996). The existing studies have proved that the resources owned by individuals can greatly promote employees creativity (Binyamin and Carmeli, 2010; Kwan et al., 2018). These resources include not only instrumental resources, but also psychological energy resources owned by individuals (Amabile, 1996; Byrne et al., 2014; Amabile and Pratt, 2016; Han et al., 2017).

First of all, the available resources of individuals are limited. Leaders' criticism, accusation and abuse will not only generate negative emotions and reduce employees' sense of efficacy and confidence, but also consume employees' existing cognitive resources to adjust and balance, resulting in the insufficient availability of individual resources (Nandkeolyar et al., 2014; Lin et al., 2015; Xu et al., 2015). Unlike other theories, the conservation of resource theory believes that individuals are more likely to protect their own resources to avoid the loss of their own energy resources and cause further pressure when faced with insufficient resources. Therefore, individuals will devote less energy resources to tasks other than task performance (creative activities), and adopt simpler cognitive strategies (Byron et al., 2010; Zhang et al., 2014a), invest less positive emotional resources to innovate and develop meaningful and innovative ideas and programs (Han et al., 2017). Secondly, proposing new ideas or ideas at work needs to break the existing and conventional procedures or methods at work. Leaders are required to provide support and feedback for innovative ideas (Scott and Bruce, 1994). However, leaders are not tolerant to employees who propose new ideas and restrict employees' access to critical work information (Xu et al., 2015; Kwan et al., 2018), which makes employees feel that they can not obtain leadership support. This is not conducive to generating new and meaningful innovative ideas. Finally, leaders' behaviors such as criticism, accusation, indifference and low tolerance of errors will reduce employees' interest in work, hinder the intrinsic motivation of innovative participation, and inhibit employee creativity (Zhang et al., 2014a). Related scholars have also studied the negative impact of abusive supervision on employee creativity from social identity, psychological safety, and social cognition. Therefore, the following hypotheses are proposed:

***H1: Abusive supervision is negatively related to employees' creativity***.

#### 2.2.2. Abusive Supervision and Employee Psychological Availability

Psychological availability is individual knowledge of the availability of physical, emotional and cognitive resources, which reflects individual ability and confidence. Firstly, stress, insecurity and distractions at work can reduce employees' psychological availability (Restubog et al., 2011). Under the long-term and continuous hostility of leaders, individuals will feel strong psychological pressure, more uncertainty and psychological insecurity (Restubog et al., 2011), which will cause individuals to worry about their own resource status at work and reduce employees' belief in acquiring physical, emotional and cognitive resources. In addition, Hirschfeld and Thomas (2008) believe that psychological availability is largely affected by the supply of individual resources.On the one hand, unlike inclusive leadership, abusive supervision represents limited superior support (Kim et al., 2016), that is to take continuous emotional and psychological hostility toward subordinates (Harvey et al., 2007), which affects employees' cognitive and physical resources (Fredrickson, 2004). On the other hand, abusive supervision as a controlled management method (Shalley et al., 2004), restricts employees access to key information (Xu et al., 2015; Kwan et al., 2018), and reduces employees' work time cognitive resources. Due to the insufficient supply of emotional resources and cognitive resources, it will inevitably reduce the psychological expectations and perceptions of employee resources, resulting in a decline in psychological availability. Some studies have also confirmed that abusive supervision will consume the psychological resources of individuals and reduce the ability and confidence of employees to obtain resources (Kim et al., 2016). Therefore, the following hypotheses are proposed:

***H2: Abusive supervision is negatively related to employees' psychological*** ***availability*.**

#### 2.2.3. Employee Psychological Availability and Creativity

Creativity is the innovative and valuable ideas, suggestions and measures proposed by employees' in the organization for existing products, services and processes. It is full of uncertainties and risks, requires employees to invest enough energy resources such as emotions, attention and patience. Psychological availability provides an important resource for individuals to engage in creative activities and express creativity. Individual creativity requires the collaborative input of cognitive resources (creating ideas), psychological resources (processing failures), and social resources (exchanging ideas with others) (Binyamin and Carmeli, 2010). Individuals have more physical, emotional, and cognitive resources that can be used, when they have higher psychological availability. Meanwhile, they will be more willing to devote additional resources to things outside of his work, such as creative activities (Galinsky et al., 2008). Sufficient cognitive resources (cognitive flexibility, richness, characterized by the integration of open mode of thinking), emotional resources (positive emotions) and participate in the work of faith and preparation, can make the individual in the face of difficulties, maintain energetic and high spirits, more sustainable and learning (Binyamin and Carmeli, 2010), and seeking more actively involved in creativity and innovation (Vinarski-Peretz and Carmeli, 2011). The theory of creative component points out that sufficient professional skills and intrinsic motivation can induce individual innovation behavior (Amabile and Pratt, 2016; Han et al., 2017; Kwan et al., 2018). Employees with high psychological availability can maintain resilience when confronted with challenges. They can actively seek ways and methods that can change the current work situation or improve work efficiency (Baer and Oldham, 2006). They also can actively seek information to generate creativity (Vinarski-Peretz et al., 2011). In addition, the individual physical, emotional and cognitive energy will increase the innovation vitality, which also helps to improve the creativity of employees (Shirom, 2003; Carmeli et al., 2014). At present, studies have also confirmed that the individual psychological availability can positively affect employees' innovation (Binyamin and Carmeli, 2010). In conclusion, this research believes that when employees have higher psychological availability, they will continue to invest more work enthusiasm in their work, contribute more intelligence and knowledge and propose more innovation and value thoughts. Therefore, we propose the following hypothesis:

***H3: Psychological availability is positively related to employees' creativity*.**

#### 2.2.4. Abusive Supervision, Psychological Availability, and Employee Creativity

Individual creative behavior needs certain working conditions, psychological state and motivation. However, motivations, and leadership styles or behaviors often need to indirectly affect subsequent behaviors by influencing employees' internal psychological state (Parker et al., 1996). Creative activities require individuals to invest large resources and energy (Byrne et al., 2014). At work, management behaviors such as continuous criticism, accusation, and insults by the leader not only release a negative signal, but also requires employees' to use more energy to manage and respond. In addition, abusive supervision will not actively care about employees' emotional and psychological needs, have a low tolerance for employees' errors, and restrict employees' access to certain key information. This will reduce employees' psychological expectations and beliefs about their own psychological resources. To cope with the continuous abuse of leaders and meet daily work needs, individuals will protect limited resources, reduce non-task performance or innovation participation and reduce employees' innovation vitality. Consequently, employees can neither fully identify problems at work nor actively introduce new working methods, which inhibits creative activities (Zhang et al., 2014a). Some researchers have shown that psychological availability, as one of the psychological conditions, can significantly mediate the impact of stress, uncertainty and leadership style on individual creativity (Binyamin and Carmeli, 2010). Based on the above discussion, the following hypotheses will be proposed:

***H4: Psychological availability mediates the relationship between abusive*** ***supervision and employees' creativity,such that the higher abusive supervision*** ***is the lower employees' creativity*.**

#### 2.2.5. The Moderating Effect of Performance Improvement Attribution

Abusive supervision is the subordinates perception of behavior. The impact of abusive supervision is not only influenced by the manager itself but also varies in individual perceptions (Tepper, 2007). The attribution of employees to this behavior will influence the psychological changes of individuals after they are subjected to abusive supervision (Burton et al., 2014; Chan and Mcallister, 2014). Compared with employees with low-performance improvement attribution, high-performance improvement attribution employees believe that leaders criticisms and accusations are for improving self-performance. The negative feedback from the leader on work performance is to encourages me to improve my work methods and complete my tasks on time. If the personal performance is very high, the leader will not deliberately criticize and accuse me, so the individual will not have negative emotions such as fear, depression. The loss of psychological resources will not be too much, and then the individual's evaluation of the availability of their own resources will not drop sharply. Vinarski-Peretz and Carmeli (2011) believes that employees' perceived concern from leaders has a positive impact on psychological availability. Therefore, when employees attribute the abusive supervision behavior of the leader to the importance of their own achievements, the individual belief in having cognitive, emotional, and physical resources will not be too low. However, employees with low-performance improvement attribution will reduce their psychological availability to a certain extent when faced with leaders criticism, accusations, and abuse. Existing studies have also confirmed that individual attribution style and active processing strategies based on task enhancement can weaken the negative impact of abusive supervision on individual emotions, cognition, and behavior (Liao et al., 2010; Mawritz et al., 2014; Nandkeolyar et al., 2014).

***H5: Performance improvement attribution moderates the relationship between*** ***abusive supervision and employees' psychological availability such that*** ***the negative relationship between abusive supervision and psychological*** ***availability is weaker when employees' performance improvement attribution*** ***is high*.**

We further believe that such that performance improvement attribution could moderate the impact of abusive on creativity *via* psychological availability. Besides, for the reason that the influence of abusive supervision could become different by the level of performance improvement attribution, we think that, under a high level of performance improvement attribution, abusive supervision have limited influence on psychological availability, which does not dramatically decreases employees' innovation. By contrast, when the attribution of performance improvement is low, psychological availability could be easily declined by abusive supervision, leading to decrease employees' creativity. Thus, taking hypothesis 4 and hypothesis 5 together, we propose:

***H6: Performance improvement attribution moderates the mediation effect*** ***of psychological availability to the relationship between abusive supervision*** ***and employees' creativity, such that the mediation effect is weaker when*** ***performance improvement attribution is high*.**

## 3. Methods and Procedures

There were no unethical behaviors in the research process, because the study focused on the influence mechanism of abusive supervision on employees' creativity and did not involve human clinical trials or animal experiments. The data were collected anonymously and the questionnaire was completed voluntarily; therefore, ethical approval and consent was also not required for this study in accordance with the local legislation and institutional requirements.

### 3.1. Data Collection

The study is a cross sectional design which refers to the collection of valid data from different individuals through questionnaire at the same time. We collected data from the employees' of different companies. The employees completed scales on abusive supervision, psychological availability, performance improvement attribution and evaluated creativity by themselves. Therefore, this study focus on the individual level in measurement and analysis. To ensure the rationality of the questionnaire and the validity of the data, we first conducted a pre-test in September 2018. The predictive test site is an artificial intelligence company (IFLYTEK CO.LTD.) in Hefei, China. We contacted company leaders to get permission to do the test. We randomly invited some employees to test the questionnaire and interviewed them. The results showed that there was no significant difference in the questionnaire among employees of different positions. And the questionnaire was modified appropriately according to the results of the interviews. From October to December 2018, we randomly sent electronic questionnaires to colleagues, working classmates and friends through WeChat, email and other Internet means (Cochran, 1979). These people are different from the members of the predictive test. We explained the content and purpose of the questionnaire to them in detail. During this period, we kept in touch with them by telephone, WeChat and other Internet means.

A total of 300 questionnaires were issued, 250 questionnaires were collected, 16 invalid questionnaires were eliminated due to data incompleteness, and 234 valid questionnaires were obtained. The effective response rate of questionnaires was 78%. Table 1 summarizes the basic information of the respondents. The respondents include 119 male (50.9%) and 115 female (49.1%). The distribution of men and women is relatively even. In terms of age structure, the proportion of young people under the age of 25 accounts for 60.7%, and the number of people with 26–30 years old make up 33.8%. According to the educational level, the respondents are mainly comprised of undergraduate and master, accounting for 95.7%. The work duration of most of the respondents under 3 years, accounting for 85.5 percent of the total sample. The study suggests that abusive supervision has a greater impact on employees with low working duration. Meanwhile, young employees' prefer to carry out creative work. Therefore, we believe that the sample distribution is reasonable.

**Table 1:** Basic information of the respondents.

**Attributes**	**Items**	**Frequency**	**Percent (%)**
Gender	Male	119	50.9
	Female	115	49.1
Age	Under 25	142	60.7
	26–30	79	33.8
	31–35	12	5.1
	36–40	1	0.4
	Over 40	0	0
Education	Junior college	4	1.7
	College	6	2.6
	Undergraduate	136	58.1
	Master	88	37.6
	Ph.D	0	0
Work duration	Under 1	114	48.7
	1–3	86	36.8
	4–5	17	7.3
	6–10	15	6.4
	Over 10	2	0.8

Following the guidelines summarized by Podsakoff et al. (2003), this study may reduce the common method variance in two ways. Firstly, we performed sufficient prediction test, and the prediction test was separated from the members and time of the questionnaire survey. Secondly, we explained the content and purpose of the questionnaire to the respondents in detail to avoid cognitive biases.

### 3.2. Measurement

According to the suggestion of Brislin (1980), we translated the scale from English to Chinese using the translation and back-translation method. In the questionnaire survey process, we asked each participant to rate the extent to which they agree with each statement by selecting a number from 1 to 5. It means that the anchor points in our questionnaire adopt the frequently used 5-level Likert type scale, in which “1” means strongly disagree, “3” means neither agree nor disagree and “5” means strongly agree.

(1) Abusive Supervision. This study uses the abusive supervision scale developed by Tepper (2000) to measure employees' perception of leadership abusive supervision behavior. The scale has a total of 15 items and is self-evaluated by employees. Sample items are, “my supervisor can laugh at me” and “my supervisor can say my idea is stupid.” The internal consistency coefficient of the scale in this study is 0.935.

(2) Performance Improvement Attribution. In this study, the employee performance improvement attribution scale developed by Liao et al. (2010) has been proven to have good reliability. The scale has 5 items in total and is self-evaluated by employees. Sample items are, “my supervisor expects higher performance from me” and “my supervisor will tell about my mistakes and problems.” The internal consistency coefficient in this study is 0.825.

(3) Psychological Availability. This study uses the psychological availability scale developed on the basis of Kahn (1990), because the scale completely includes the individual's perception and evaluation of the availability of physical resources, emotional resources, and cognitive resources. The scale has 5 items in total and is self-evaluated by employees. Sample items are, “I believe I am capable of dealing with challenging work” and “I believe I can deal with the problems in my work.” The internal consistency coefficient of the scale in this study is 0.887.

(4) Creativity. This study uses a scale developed by Tierney and Farmer (2002), which has a total of 4 items and is self-evaluated by employees. Sample items are, “I can be the first to try out new ideas or methods” and “I can actively seek new ways or new ideas to solve the problem.” The internal consistency coefficient of the scale in this study is 0.856.

(5) Control Variable. The research conclusions of some scholars have confirmed that the employee's gender, age, education level and working duration have a significant impact on the employee's creative behavior (Janssen and Huang, 2008; Furnham and Nederstrom, 2010). Therefore, we choose gender and age, education level and working duration as control variables, and study of the impact of core variables on employees' creativity.

## 4. Data Analysis

### 4.1. Common Method Variance

All answers were collected from a single source, thus common method variance could be a threat to the validity of this study (Podsakoff et al., 2003). To reduce the common method variance of the data, this study uses the Harman single factor test to performed the common method deviation bias by SPSS 22 (Podsakoff and Organ, 1986). The test results show that the exploratory factor analysis is used to extract a principal component and the variance explanation rate is 31.839%, which is lower than 40%. Therefore, it can be considered that there is no common method variance in the data.

### 4.2. Reliability and Validity Test

The reliability of the data was analyzed by Mplus 7.4 and SPSS 22. Using confirmatory factor analysis, we obtain the standardized factor load of each latent variable, and calculate the CR and AVE of each variable. As shown in Table 2, all of the variables Cronbach's α are above 0.8. The composite reliability (CR) values of all the constructs are more than 0.7. Therefore, the results in Table 2 indicate that the scale has a high degree of reliability. In addition, most of the variables AVE are above 0.5 except the AVE of the abusive supervision is 0.498. The result shows that each item has a strong degree of interpretation of the construct, so we believe that the convergence validity of each scale is better.

**Table 2:** Reliability results.

**Variable**	**Cronbach's α**	**CR**	**AVE**
Abusive supervision	0.935	0.937	0.498
Psychological availability	0.887	0.891	0.622
Creativity	0.856	0.862	0.610
Performance improvement attribution	0.825	0.832	0.502

The following metrics were used to assess the fitness of model: chi-square/degrees of freedom (λ^2^/df), goodness-of-fit index (GFI), adjusted goodness-of-fit index (AGFI), normed fit index (NFI), comparative fit index (CFI), and root mean square of error approximation (RMSEA). According to Scott and Judy (1995), the λ^2^/df of a fit model should be less than 3.0, CFI and TLI should be greater than 0.9. The root mean square error of approximation (RMSEA) should be below the recommended range of acceptability (0–0.05) recommended by Maccallum et al. (2006). As shown in Table 3, the fitness of four-factor model is better than others. Therefore, the four-factor model has the best fit, indicating that the overall scale has better discriminative validity.

**Table 3:** Confirmatory factor analyses.

**Model**	**Factor**	**λ^2^**	**df**	**λ^2^/df**	**RMSEA**	**SRMR**	**TLI**	**CFI**
Four-factor Model	AS;PA;C;PIA	157.284	113	1.392	0.041	0.045	0.976	0.980
Three-factor Model	AS;PA+C;PIA	282.451	116	2.435	0.078	0.054	0.914	0.926
Three-factor Model	AS+PA;C;PIA	682.339	116	5.882	0.144	0.111	0.706	0.749
Three-factor Model	AS;PA;C+PIA	553.465	116	4.771	0.124	0.123	0.783	0.815
Two-factor Model	AS+PA+C;PIA	803.842	118	6.812	0.158	0.115	0.650	0.696
Two-factor Model	AS;PA+C+PIA	663.525	118	5.623	0.141	0.129	0.722	0.758
Two-factor Model	AS+PA;C+PIA	1042.613	118	8.836	0.183	0.152	0.528	0.591
One-factor Model	AS+PA+C+PIA	1174.610	119	9.871	0.195	0.158	0.466	0.533

*AS refers to abusive supervision; PA refers to psychological availability; C refers to creativity; PIA refers to performance improvement attribution*.

### 4.3. Descriptive Analysis

The mean, standard deviation and correlation coefficient of each variable analyzed by SPSS 22, as shown in Table 4. It can be seen from Table 4 that the abusive supervision are significantly negative correlated with psychological availability (*r* = −0.312, *p* < 0.01) and creativity (*r* = −0.302, *p* < 0.01), which initially verified Hypothesis 1 and 2. There is a significant positive correlation between psychological availability and creativity (*r* = 0.684, *p* < 0.01). Hypothesis 3 is initially verified.

**Table 4:** Means, standard deviations, and interrelations of variables.

**Variable**	**MEAN**	**SD**	**1**	**2**	**3**	**4**
1. Abusive supervision	2.176	0.729	**(0.706)**			
2. Psychological availability	3.876	0.649	−0.312^**^	**(0.789)**		
3. Creativity	3.765	0.567	−0.302^**^	0.684^**^	**(0.781)**	
4. Performance improvement attribution	3.608	0.714	0.142^*^	0.242^**^	0.244^**^	**(0.709)**

* *p < 0.05*,

** p < 0.01, and ***p < 0.001. The diagonal lines represent the square root of AVE. The bold values represent the square root of AVE.

## 5. Hypothesis Testing

Mediating effect is the effect of the independent variable on the dependent variable through the intermediary variable (Baron and Kenny, 1986). To test the mediating effect of psychological availability, we followed the examination proposed by Baron and Kenny (1986). It can be seen from Table 5 that Model 1a reflects that the degree of explanation of the control variables for creativity is 4.1%. After adding independent variables in Model 2a, the regression coefficient of abusive supervision on employees creativity is significant (β = −0.311, *p* < 0.001), and the amount of R^2^ explanation increases significantly (△R^2^ = 9.4%, *p* < 0.001). It can be concluded that hypothesis 1 is supported. Model 3a shows that abusive supervision has a significant regression on employee psychological availability (β = −0.325, *p* < 0.001), and the explanatory value of R^2^ increase significantly (△R^2^ = 10.4%, *p* < 0.001), thus hypothesis 2 is supported. Model 4a shows that the regression of psychological availability on creativity is significant (β = 0.676, *p* < 0.001), and the explanatory value of R^2^ increase significantly (△R^2^ = 44%, *p* < 0.001), thus hypothesis 3 is supported. According to model 2a, abusive supervision negatively affects employees' creativity, and explains the variation of creativity by 13.5%. Model 5a shows that abusive supervision and psychological availability are introduced into regression simultaneously to verify the impact of both on creativity. Comparing Model 2a and Model 5a, after the introduction of psychological availability, the coefficient and significance of the impact of abusive supervision on employee creativity decreased (β = −0.311, *p* < 0.001; β = −0.102, *p* < 0.05). However, abusive supervision and psychological availability have a significant impact on employee' creativity. Meanwhile, compared with Model 2a, the variance of explaining creativity by abusive supervision and psychological availability was significantly increased in Model 5a (△R^2^ = 0.355). We also followed Hayes et al. (2007) suggestion to use bootstrapping method. The result reveals from Table 6 that the indirect effect is significant. The Sobel test confirmed the existence of mediation (indirect effect = −0.210, standard error = 0.036, *z* = −6.09, *p* < 0.001). Therefore, the psychological availability part mediates the relationship between abusive supervision and creativity, and hypothesis 4 is supported.

**Table 5:** The mediating role of psychological availability.

**Variable**	**Creativity**		**Psychological availability**	**Creativity**	
	**Model 1a**	**Model 2a**	**Model 3a**	**Model 4a**	**Model 5a**
Gender	−0.122	−0.148^*^	−0.087	−0.081	−0.092
Age	−0.113	−0.123	−0.148	−0.020	−0.027
Education	0.183^*^	0.174^*^	0.191^**^	0.048	0.051
Work duration	0.15	0.116	0.058	0.087	0.079
Abusive supervision		−0.311^***^	−0.325^***^		−0.102^*^
Psychological availability				0.676^***^	0.642^***^
R^2^	0.041	0.135	0.14	0.481	0.49
△R^2^		0.094^***^	0.104^***^	0.44^***^	0.355^***^
F	2.442^*^	7.130^***^	7.395^***^	42.262^***^	36.364^***^

* *p < 0.05*,

** *p < 0.01, and*

*** *p < 0.001*.

**Table 6:** The mediating effect.

**Effect**	**Path**	**Estimate**	**S.E**.	**Confidence interval 95%**
Total Effect	AS → Creativity	−0.311	0.065	[−0.411, −0.199]
Indirect Effect	AS → PA → Creativity	−0.208	0.046	[−0.282, −0.134]
Direct Effect	AS → Creativity	−0.102	0.051	[−0.182, −0.018]

Baron and Kenny (1986) believed the moderator affects the strength of the relationship between an independent variable and a dependent variable. This study uses a three-step test method of adjustment regression analysis to test the moderating effect of performance improvement attribution. Before regression, the relevant variables were standardized. From Table 7, Model 3b shows that the interaction coefficient of abusive supervision and performance improvement attribution is significant (β = 0.161, *p* < 0.01), and can significantly explain the additional 2.4% of the variation (△R^2^ = 0.024, *p* < 0.01), thus hypothesis 5 is supported. In other words, the negative impact of abusive supervision on psychological availability was weakened for employees with a higher attribution of performance improvement.

**Table 7:** The moderating effect of performance improvement attribution.

**Variable**	**Psychological availability**		
	**Model 1b**	**Model 2b**	**Model 3b**
Gender	−0.060	−0.118^*^	−0.119^*^
Age	−0.138	−0.106	−0.103
Education	0.201^**^	0.156^*^	0.147^*^
Work duration	0.093	0.021	0.008
Abusive supervision		−0.368^***^	−0.372^***^
Performance improvement attribution		0.289^***^	0.337^***^
Abusive supervision ^*^ Performance improvement attribution			0.161^**^
R^2^	0.036	0.219	0.243
△R^2^		0.183^***^	0.024^**^
F	2.166	10.624^***^	10.356^***^

* *p < 0.05*,

** *p < 0.01, and*

*** *p < 0.001*.

When performance improvement attribution takes two different condition values, that is, the mean plus one standard deviation and the mean minus one standard deviation, the indirect effect of abusive supervision on employees' creativity through employee psychological availability is significantly different. It can be seen from Table 8 that under the high-performance improvement attribution level, the estimated effect of abusive supervision on the psychological availability of employees is −0.206, with a 95 percent CI [−0.315, −0.0.098], excluding zero. Under the low-performance improvement attribution level, the estimated effect of abusive supervision on the psychological availability of employees' is −0.457, with a 95 percent CI [−0.599, −0.292], excluding zero. The difference between the two effects is 0.251. Therefore, performance improvement attribution can significantly regulate the relationship between abusive supervision and psychological availability, hypothesis 5 is supported again. The results in Table 8 also confirm that the influence of leadership abusive supervision on employees creativity through psychological availability is regulated by the employee's attribution level. Specifically, if the performance improvement attribution level is high, the impact of abusive supervision on creativity through psychological availability is −0.105, with a 95 percent CI [−0.173, −0.055], excluding zero.If the performance improvement attribution level is low, the impact of abusive supervision on creativity through psychological availability is −0.234, with a 95 percent CI [−0.326, −0.142], excluding zero. Compared with employees' with low-performance improvement attribution levels, high-performance improvement attribution employees can feel less loss of creativity. The hypothesis 6 is supported.

**Table 8:** The moderated mediating effect.

	**First stage: AS → PA**		**Second stage: PA → Creativity**	
	**Estimated**	**Confidence interval 95%**	**Estimated**	**Confidence interval 95%**
High PIA	−0.206	[−0.315,−0.0.098]	−0.105	[−0.173,−0.055]
Low PIA	−0.457	[−0.599,−0.292]	−0.234	[−0.326,−0.142]
Difference	0.251	[0.060,0.43]	0.129	[0.031,0.238]

**Figure 2:**
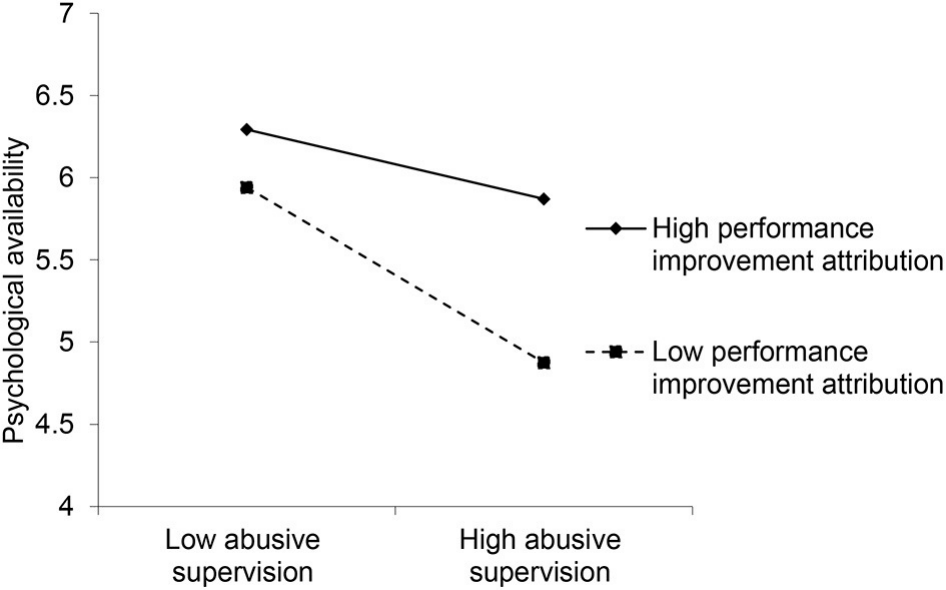
Moderating effect of performance improvement attribution on relationship between abusive supervision and psychological availability.

By drawing the adjustment effect diagram, the adjustment effect can be judged more clearly. It can be seen from Figure 2 that the regulating effect graph presents a trend to the lower right, that is, abusive supervision has a negative impact on the psychological availability of employees'. In addition, compared with employees with low-performance improvement attributions, employees with high-performance improvement attributions can feel less psychological availability decline.

## 6. Discussion and Conlusions

**Figure 3:**
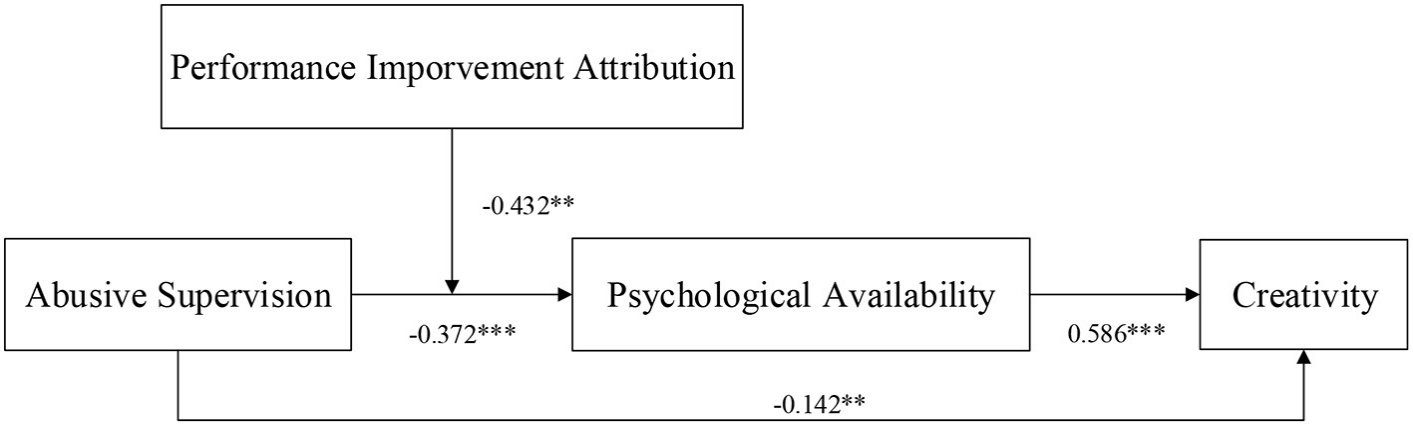
The moderated mediating model *p < 0.05, **p < 0.01, and ***p < 0.001.

The present study investigates how abusive supervision affects employees' creativity through psychological availability. In the event, we found that abusive supervision have negative effect on employees' creativity. Psychological availability plays a partial mediating role between abusive supervision and creativity. Furthermore, performance improvement attribution can weaken the effect of abusive supervision on creativity *via* employees' psychological availability (Figure 3). As our results showed, the mediating effect of abusive supervision on employee creativity through psychological availability is weaker when employees with high-performance improvement attribution than among employees with low-performance improvement attribution.

### 6.1. Theoretical Implications

Our study has contributed to the literature in multiple ways. Firstly, this study has verified the negative impact of abusive supervision on employees' creativity, and provides a new perspective enhancing our understanding of mechanisms of abusive supervision and creativity. The results show that the abusive supervision has a negative impact on employees' creativity (Liu et al., 2012). On the one hand, the leadership's actions such as criticism, accusation, and abuse will consume employees' psychological resources, resulting in insufficient availability of resources (Zhang et al., 2012). Based on the intention of resource conservation, individuals will devote most of their energy to maintaining their remaining resources, which will eventually make employees unable to devote themselves to work, resulting in a reduction in employee creativity. On the other hand, the leader does not support new ideas proposed by employees, and restricts information exchange between employees, making employees feel that they have lost the support of their superiors. They will not work efficiency and other activities that may bring interpersonal risks.

Second, this study introduced employee's psychological availability as an intermediary variable and enriches the literature research about creativity (Byron et al., 2010; Nandkeolyar et al., 2014). This study proves that psychological availability can significantly transmit the negative impact of abusive supervision on employees' creativity (Binyamin and Carmeli, 2010). At work, the higher the psychological availability is, the richer the physical, emotional and cognitive resources that an individual can mobilize. Based on the conservation of resource theory, employees' will devote extra time and energy to activities outside the work, such as work innovation. The study also verified that abusive supervision reduces employees' psychological security, increases their uncertainty, consumes employees' energy resources, and thereby reduces employees' psychological availability. In addition, psychological availability plays a partially mediating role in the impact of abuse supervision on creativity, which also shows that abusive supervision can affect employees' creativity through other mediating variables.

Third, the research examined the moderating effect of attribution of performance improvement attribution. Although abusive supervision can reduce the level of resource availability of employees by consuming employees' cognitive resources, the extent of its effects is often affected by certain individual differences, such as attribution. Individuals with different attribution tendencies have different interpretations of leadership behavior. Compared with individuals with low-performance improvement attribution, individuals with high-performance improvement attribution are more inclined to explain leadership behavior in a positive way (Martinko et al., 2011, 2012). The research results also indirectly verify that performance improvement attribution, as a positive individual difference, will compensate for the consumption of individual resources and weaken the impact of negative behaviors on the availability of psychological energy for employees.

### 6.2. Managerial Implications

Based on results of the research, the following useful suggestions to business managers are put forward. First, managers should improve management methods and cultivate positive leadership behaviors. For example, managers should be trained to reduce the pressure and distress caused by negative leadership behaviors on employees (Gonzalez-Morales et al., 2018). They should communicate with subordinates positively, and enhance employees' passion for work and broadening their cognitive scope to do more valuable work. Second, managers should pay attention to the care and guidance of employees' emotions and cognition. Shirom (2007) pointed out that employees' resources such as physiology resources, emotion resources and cognition resources contribute to individual creative behavior. The company should encourage leaders to care about the emotional and psychological needs of subordinates, recognize subordinates, and enrich employees' emotional resources. Managers should provide staff with timely and effective feedback and personalized career development plans to increase employees' cognitive resources. Third, a company should cultivate a positive organizational atmosphere and guide employees to establish a sense of positive attribution. The managers should strengthen cultural construction in the company and promote simple and sincere communication culture. It will guide employees to bravely point out the inappropriateness of these behaviors when facing criticism from leaders.

### 6.3. Limitations and Future Research

There are some limitation to this study, which must be addressed in future. First,the research do not consider organizational factors such as the company or team, and we will build a multi-layer model for further discussion. Second, the age of most of the respondents in this study is <30 years. The duration of working experience is limited to <3 years. The distribution of age groups and working duration is uneven. In addition, there are other control variables such as work environment, colleagues' attitudes, self-efficacy, which are not involved in this study. In the future, we could further analyze these control variables and conduct comparative study between different groups.

## Data Availability Statement

The original contributions presented in the study are included in the article/supplementary material, further inquiries can be directed to the corresponding author/s.

## Ethics Statement

There were no unethical behaviors in the research process, because the study focused on the influence mechanism of abusive supervision on employees' creativity and did not involve human clinical trials or animal experiments. The data were collected anonymously and the questionnaire was completed voluntarily, therefore ethical approval and consent was also not required for this study in accordance with the local legislation and institutional requirements.

## Author Contributions

XZhan and YW developed the conceptual framework and revised the whole paper. CW analyzed the data and wrote the paper. XZhao and YP collected the data and discussed the results. All authors contributed to the article and approved the submitted version.

## Conflict of Interest

The authors declare that the research was conducted in the absence of any commercial or financial relationships that could be construed as a potential conflict of interest.
